# Distinct Autoimmune Anti-α-Synuclein Antibody Patterns in Multiple System Atrophy and Parkinson’s Disease

**DOI:** 10.3389/fimmu.2019.02253

**Published:** 2019-09-24

**Authors:** Jonas Folke, Rasmus Rydbirk, Annemette Løkkegaard, Lisette Salvesen, Anne-Mette Hejl, Charlotte Starhof, Sára Bech, Kristian Winge, Søren Christensen, Lars Østergaard Pedersen, Susana Aznar, Bente Pakkenberg, Tomasz Brudek

**Affiliations:** ^1^Research Laboratory for Stereology and Neuroscience, Bispebjerg-Frederiksberg Hospital, University Hospital of Copenhagen, Copenhagen, Denmark; ^2^Department of Neurology, Bispebjerg-Frederiksberg Hospital, University Hospital of Copenhagen, Copenhagen, Denmark; ^3^Novo Nordisk Foundation, Hellerup, Denmark; ^4^Bispebjerg Movement Disorders Biobank, Bispebjerg-Frederiksberg Hospital, University Hospital of Copenhagen, Copenhagen, Denmark; ^5^H. Lundbeck A/S, Copenhagen, Denmark; ^6^Department of Immunology and Microbiology, Faculty of Health, University of Copenhagen, Copenhagen, Denmark; ^7^Institute of Clinical Medicine, Faculty of Health, University of Copenhagen, Copenhagen, Denmark

**Keywords:** autoimmunity, antigens, autoantibodies, neurology, plasma, alpha-synuclein, multiple system atrophy, parkinson's disease

## Abstract

Aggregation of alpha-synuclein (α-syn) is considered to be the major pathological hallmark and driving force of Multiple System Atrophy (MSA) and Parkinson's disease (PD). Immune dysfunctions have been associated with both MSA and PD and recently we reported that the levels of natural occurring autoantibodies (NAbs) with high-affinity/avidity toward α-synuclein are reduced in MSA and PD patients. Here, we aimed to evaluate the plasma immunoglobulin (Ig) composition binding α-syn and other amyloidogenic neuropathological proteins, and to correlate them with disease severity and duration in MSA and PD patients. All participants were recruited from a single neurological unit and the plasma samples were stored for later research at the Bispebjerg Movement Disorder Biobank. All patients were diagnosed according to current consensus criteria. Using multiple variable linear regression analyses, we observed higher levels of anti-α-syn IgG1 and IgG3 NAbs in MSA vs. PD, higher levels of anti-α-syn IgG2 NAbs in PD compared to controls, whereas anti-α-syn IgG4 NAbs were reduced in PD compared to MSA and controls. Anti-α-syn IgM levels were decreased in both MSA and PD. Further our data supported that MSA patients' immune system was affected with reduced IgG1 and IgM global levels compared to PD and controls, with further reduced global IgG2 levels compared to PD. These results suggest distinct autoimmune patterns in MSA and PD. These findings suggest a specific autoimmune physiological mechanism involving responses toward α-syn, differing in neurodegenerative disease with overlapping α-syn pathology.

## Introduction

Multiple system atrophy (MSA) and Parkinson's disease (PD) are neurodegenerative movement disorders which share many clinical and pathomorphological features (1, 2). The pathological hallmark for these two diseases is the aggregation of the protein α-synuclein (α-syn), which precipitates as glial cytoplasmic inclusions in oligodendroglia in MSA and neuronal Lewy body inclusions in PD (1). Furthermore, α-syn is also present in large amounts in the blood of both patients and healthy individuals and as inclusions in the enteric nervous system in PD (3, 4). MSA is considered a sporadic disease of uncertain etiology, whereas at least 90% of PD cases are idiopathic and ~10% represent rare Mendelian hereditary forms (1). Discriminating between MSA and idiopathic PD can be difficult, especially at early stages, but usually MSA presents a more pernicious course. Advanced molecular imaging can, to some degree, aid to distinguish PD from MSA and other atypical parkinsonism syndromes (5), but definite diagnoses is only possible through post-mortem examination (6). Hence, there is a need for more convenient and sensitive biomarkers for the disorder, which has great importance for prognostic measures and development of therapeutic strategies.

Naturally occurring autoantibodies (NAbs) are components of the innate immune system present throughout life. NAbs recognize and bind self-antigens without prior immunization (7). They are distinct from adaptive antibodies, which are highly specific to exogenous pathogens to which the host has experienced previous exposure. NAbs are predominantly of IgM and IgG classes. Their main functions are maintenance of physiological and immune homeostasis by, e.g., removing aging cells, tumor cells, cellular debris, and altered self-molecules, to inactivate cytokines, and to mask autoantigens (8). In recent years, it has been suggested that IgMs constitute the first line of defense against neoepitopes as reviewed by (9). It is further believed that IgM NAbs are spontaneously produced by memory B-1 cells and upon activation through the B cell receptor, memory B cell proliferate and a majority Ig class-switches e.g., IgG subclasses (10, 11). Quantitative and functional differences in specific autoantigenic NAbs and/or changes of NAb profiles over time within individuals have been associated with various pathophysiological conditions (12, 13), including neurodegenerative diseases (14). Indeed, a vast number of studies have investigated α-syn specific NAbs in PD, often with contradictory results. The majority of studies have found unchanged anti-α-syn NAb levels (15–19) and increased levels in early PD (15, 20–24). One study reported reduced anti-α-syn NAb levels in PD (25). The only study reporting plasma levels of anti-α-syn NAbs in atypical parkinsonism disorders reported no differences in patients in a combined group including MSA, Lewy body Dementia, another synucleinopathy, and Progressive Supranuclear Palsy, a tauopathy, compared to controls and PD (17). Furthermore, Orr et al. (26) showed that IgG deposits co-localized with α-syn pathology in PD brains, suggesting an induced pathological α-syn response. The inconsistency of previous studies probably results from limitations in reporting total levels of immunoglobulin (Ig)G autoantibodies, without differentiating into α-syn reactive IgG subclasses and IgM. To this date, no studies have evaluated levels of IgG subclasses and IgM NAbs against any amyloidogenic proteins in a pure MSA patient group and in PD patients.

Recently, we reported that the repertoire of high-affinity/avidity IgG autoantibodies against α-syn was significantly reduced in PD and almost absent in MSA (27). This implies impaired capacity for immune clearance and/or blocking of toxic α-synuclein species, which may reflect the distinct pathology and symptom progression patterns in the two disorders. Based on these results we hypothesized that the distributions of IgG subclasses, i.e., IgG1-IgG4, total IgG and IgM, specific toward α-syn, may show different α-syn autoimmune responses in MSA and PD vs. controls. Moreover, we hypothesized that the humoral aberrancy is confined to α-syn and to further examine whether alterations are a general amyloidogenic tendency, we investigated autoantibody subclass levels toward serine-129-phosphorylated α-syn, and the closely related β- and γ-syn, together with plasma levels of the dementia-associated amyloidogenic proteins amyloid-β (Aβ) and tau. The levels of NAbs were additionally correlated with disease severity and disease duration.

## Methods

### Subjects and Samples

Plasma samples from patients with probable MSA (*n* = 34), PD (*n* = 43), as well as control subjects (*n* = 59) were obtained from the Bispebjerg Movement Disorder Biobank, Department of Neurology, Bispebjerg-Frederiksberg Hospital, University Hospital of Copenhagen, Copenhagen, Denmark. These were all outpatients on ambulatory visit from August 2007 to September 2015 that had a blood sample taken for inclusion in the Bispebjerg Movement Disorder Biobank. Patients were followed by a Movement Disorder specialist. Patients at recruitment time were given either a “possible” or a “probable” diagnosis of MSA and PD according to consensus criteria (28, 29). Only individuals that progressed from possible diagnosis at recruitment to probable MSA diagnosis at the follow-up visit (after 2015) were included in this study. Healthy control subjects were selected from the same biobank based on the absence of any immunological diseases or history of any neurological disorders including head trauma. Individuals receiving immunomodulatory treatment were not chosen for the study. All individuals in this study were Caucasian, with a few exceptions. The preanalytical handling was carried out according to previous recommendations for biobanking except the centrifugation temperature was set at 5°C (30). Briefly, venous whole blood was drawn to 7.5 mL EDTA tubes (Monovette, Sarstedt, Germany, cat# 01.1605.001) and centrifuged at 2,000 × g for 10 min at 5°C within 30 min of collection. The plasma fraction was aliquoted in 400 μl polypropylene cryotubes and stored at −80°C within 90 min. The storage time between samples did not differ between MSA patients [mean = 5.7 years; standard deviation (SD) = 2.3], PD patients [mean = 5.2; SD = 0.7], and controls [mean = 5.5; SD = 0.2]. Further, the measured outcomes did not correlate with the storage time (Pearson's *r*; *p* > 0.05). Demographic data are summarized in Table 1. Age upon collection of the samples was significantly lower in controls subjects compared to MSA and PD patients (*p* < 0.001), and sex ratio differed significantly between PD and controls (*p* = 0.011). Further, as expected, disease duration was significantly longer in PD (*p* = 0.03) and MSA patients had more advanced disease severity (*p* < 0.001), evaluated by Hoehn & Yahr (H & Y) staging (31). Hence, to avoid confounding, all statistical analyses were adjusted for age and sex.

**Table 1:** Demographic and clinical data.

	**Controls** **(*n* = 59)**	**MSA-C patients** **(*n* = 11)**	**MSA-P patients** **(*n* = 20)**	**MSA-C+P patients** **(*n* = 3)**	**MSA patients total (*n* = 34)**	**PD patients** **(*n* = 43)**	***p*-values**
Male/female^#^	10/49	3/8	9/11	0/3	12/22	30/13	0.011^*^
Age, years^*Ψ*^	41.2 (13.5) [20-85]	65 (7.1) [52-77]	65.4 (10.1) [46-79]	60.7 (10.7) [49-70]	64.8 (9.1) [46-79]^**§**^	62.8 (6.8) [46-78]^**§**^	<0.001^*^
Age of onset^$^	–	57.6 (6.9) [42-67]	60 (10.4) [43-78]	55 (16.4) [37-69]	58.9 (9.8) [37-78]	54.8 (9.1) [37-74]	0.063
Disease duration, years^$^	–	7.3 (6.5) [1-25]	5.2 (5.7) [0–22]	5.7 (5.6) [1-12]	5.9 (4.5) [0–25]	7.9 (5.3) [1-22]^**¤**^	0.031^*^
Hoehn & Yahr staging^≠^	–	3.5 (1.0) [2-5]	3.1 (0.5) [2-4]	3.7 (0.6) [3-4]	3.3 (0.7) [2-5]	2.0 (0.26) [1.5–3]^**¤**^	<0.001^*^

Results are presented as mean ± standard deviation [range]. MSA, Multiple System Atrophy; MSA-P, MSA with predominantly parkinsonian signs; MSA-C, MSA with predominantly cerebellar signs; MSA-C+P, MSA with both parkinsonian and cerebellar signs. PD, Parkinson's disease. All statistical analyses between MSA and PD were performed on total MSA patients.

# Chi-squared test.

Ψ One-way ANOVA with combined MSA group.

$ Student's t test.

≠ Mann-Whitney U-test.

* Significant p-value below 0.05.

§ Significantly different from controls.

¤ *Significantly different from combined MSA*.

### Measurement of Antigen-Specific Autoantibodies

Initially, we investigated levels of antibodies toward the pathological protein, α-syn. Moreover, to evaluate for the specificity of the binding to α-syn, which is considered to be an amyloidogenic protein, we included several other proteins with similar properties. Hence, we measured the relative levels of autoantibodies against full-length α-syn (rPeptide, USA, cat# S-1001), beta(β)-syn (rPeptide, USA, cat# S-1003), gamma(γ)-syn (rPeptide, USA, cat# S-1007), tau (2N4R) (rPeptide, USA, cat# T-1001), and amyloid-β (1-42) (rPeptide, USA, cat# A-1002) using indirect ELISA setups developed in-house. Autoantibody levels toward Phosphorylated α-syn was measured as described earlier (27). In details: (1) 96-well polystyrene plates (Nunc Maxisorp, cat# 144531) were coated with 50 μl portions of recombinant protein monomers at a concentration of 5 μg/ml (for tau: 1 μg/ml) in ice-cold 0.1 M carbonate buffer at pH 9.4 for minimum 12 h at 4°C. (2) The plates were then emptied and blocked with 3% bovine serum albumin (BSA) fraction V (Sigma-Aldrich, cat# 10735094001) containing 0.1% Tergitol^TM^ solution (Sigma-Aldrich, cat# NP40S) in phosphate-buffered saline (PBS) at pH 7.4 for 2 h at room temperature (RT). Wells were washed in five consecutive washing steps with 300 μl/well of PBS+0.05% Tween-20 (Sigma-Aldrich, cat# P1379) using a WellWash instrument (Thermo Scientific, USA). (3) Diluted plasma samples (1:100) in PBS containing 0.1% BSA were added to the plates and incubated for 1 h at RT. For IgG4 assays, plasma samples were diluted 1:50. (4) After a subsequent washing step, the plates were incubated at RT for 2 h with 50 μl of biotinylated secondary antibodies at the following concentrations: goat anti-human total IgG 1:30,000 (Sigma-Aldrich, cat# SAB3701279, RRID:AB_2783655), goat anti-human IgM (1:5,000; Sigma-Aldrich, cat# B1265, RRID:AB_258514), mouse anti-human IgG1 (1:1,000; Thermo Fisher Scientific, Cat# MH1515, RRID:AB_2539710), mouse anti-human IgG2 (1:5,000: Sigma-Aldrich, cat# B3398, RRID:AB_258546), mouse anti-human IgG3 (1:500; Sigma-Aldrich, cat# B3523, RRID:AB_258549), and mouse anti-human IgG4 (1:200; Sigma-Aldrich, cat# B3648, RRID:AB_258555). After washing, 50 μl portions of streptavidin-peroxidase (1:10,000; Sigma-Aldrich, cat# 5512) were added to each well and incubated for 30 min at RT. (5) The plates were washed for the last time before the enzymatic reaction was developed by adding 50 μl of tetramethylbenzidine (TMB) Liquid Peroxidase Substrate (Sigma-Aldrich, cat# T8665) and incubated in dark for 30 min at RT. (6) The enzymatic reaction was terminated by addition of 0.5 N Sulfuric Acid (Merck, cat# 109073), and (7) the optical density (OD) was measured on a Multiscan^TM^ FC Microplate reader (Fisher Scientific^TM^) at 450/620 nm. Before final assays, extensive optimization of each ELISA setup was done. Titer analyses of each antibody was performed, which included analyses of the positive controls on each plate to ensure homogeneity between plates, yielding %CVs ranging from 5.0 to 7.6%. Moreover, titer analyses were performed on coating and pooled plasma samples for each separate experimental setup. We also undertook Z'-factor analyses with Z'-factor values ranging from 0.77 to 0.88. Moreover, All sample ODs were normalized to a positive calibration control in a 2-fold serial dilution with a primary antibody against coated proteins/antigens and detected with a secondary biotinylated antibody specific for the primary antibody, i.e., rabbit (1:1,000; Vector, cat# BA-1000, RRID:AB_2313606) or mouse (1:1,000; Vector, cat# BA-9200, RRID:AB_2336171). The antibodies for the positive controls were mouse anti-human α-synuclein (Abcam, cat# ab27766, RRID:AB_727020), mouse anti-human β-synuclein (Abcam, cat# ab167607, RRID:AB_2783654), mouse anti-human γ-synuclein (Abcam, cat# ab89462, RRID:AB_2042990), rabbit anti-human phospho-α-synuclein Ser129 (Santa Cruz, cat# Sc-135638, RRID:AB_2302265), rabbit anti-Tau (Dako, cat# A0024, RRID:AB_10013724), and mouse anti-human amyloid-beta (Abcam, cat# ab2539, RRID:AB_303141).

### Measurement of Global Antibody Plasma Levels

To assess whether the global humoral immune system encompassed the antigen-specific IgG subclass and IgM responses, we measured the global concentrations of IgG subclass and IgM antibodies in plasma samples. We have used the commercially available Ready-SET-Go!® ELISA kits (Thermo Scientific Affymetrix eBioscience) following the manufacturer's instructions. In brief, plasma samples were diluted in the assay buffers supplied with the kits according to manufacturer-recommended: IgG1 ELISA kit: 1:2,000 (cat# 88-50560, RRID:AB_2574896); IgG2 ELISA kit: 1:500,000 (cat# 88-50570, RRID:AB_2574897); IgG3 ELISA kit: 1:40,000 (cat# 88-50580, RRID:AB_2574898); IgG4 ELISA kit: 1:1,000 (cat# 88-50590, RRID:AB_2574899); IgG-total ELISA kit: 1:500,000 (cat# 88-50550, RRID:AB_2574891), and IgM 1:20,000 (cat# 88-50620, RRID:AB_2574912).

### Determination of α-Syn Concentration

To evaluate the potential effect of anti-α-syn NAbs on peripheral α-syn levels, we measured total concentrations of α-syn in plasma samples using the commercially available U-PLEX® Human α-syn Kit (MSD® MULTI-ARRAY Assay System, cat# K151WKK) following the manufacturer's instructions. Prior the assessment of the individual samples, we optimized the assay for greatest yield using titer analyses in 1:50 diluted plasma. Plasma samples were diluted 1:50 in Diluent 49 provided by the manufacturer. The plates were read using an MSD Sector Imager S600.

### Statistical Analyses

The data analyses were performed using R v. 3.5.2 (32), GraphPad Prism v. 7.02 (GraphPad Software Inc., USA) and SPSS v. 24.0 (IBM, USA). Demographic differences were tested using one-way ANOVA, unpaired Student's *t*-test, Mann-Whitney *U*-test, and the chi-squared test. Normality was assessed using the D'Agostino Omnibus test and Shapiro Wilk test; if a normality test failed, the data were log_10_-transformed. For group comparisons, we used multiple linear regression modeling in R including the covariates age and sex with contrast for group using *Anova* from the *car* package (33). For multiple comparisons, the *glht* and *mcp* functions from the *multcomp* package (34) were applied to include covariates in *post hoc* testing using Tukey's range test. Correlation analyses were assessed using Spearman's Rank-Order correlation. Differences were considered significant at *p* < 0.05. To compensate for potential Type I error for multiple comparison of multiple linear regression modeling's, Bonferroni correction was performed with a significance cut-off at *p* < 0.0013.

## Results

### NAbs Against Pathological Antigens

**Figure 1:**
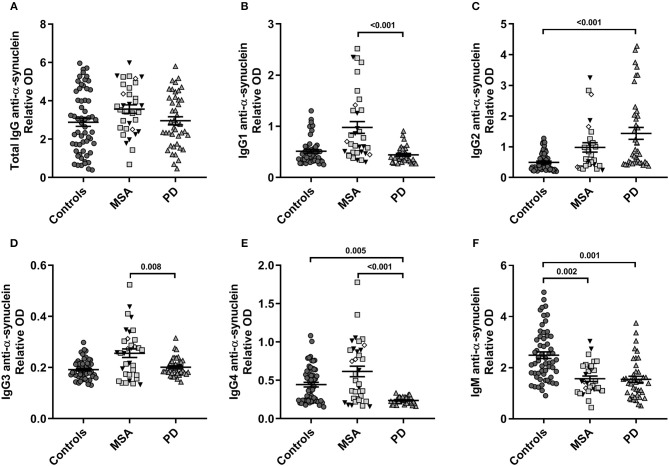
Plasma α-synuclein naturally occurring autoantibody levels. Distribution of relative anti-α-synuclein naturally occurring autoantibody plasma levels in patients with Multiple System Atrophy (MSA, *n* = 34) divided into subtypes (MSA-P: gray squares, *n* = 20; MSA-C: black triangles, *n* = 11; MSA-C+P, *n* = 3: white rhombi), patients with Parkinson's disease (PD, *n* = 34), and controls (*n* = 59). ELISA relative ODs of anti-α-synuclein **(A)** total IgG, **(B)** IgG1, **(C)** IgG2, **(D)** IgG3, **(E)** IgG4, and **(F)** IgM autoantibodies. Dot plots show relative ODs with mean values (horizontal bars) ± SEM. Differences were tested using one-way ANOVA and Tukey's *post hoc* test adjusted for age and sex.

Our model described significant differences in levels of α-syn reactive antibodies for all IgG subclasses and IgMs [IgG1: *F*_(4, 109)_ = 10.3, *p* = 3.9E-07; IgG2: *F*_(4, 114)_ = 8.4, *p* = 5.5E-06; IgG3: *F*_(4, 120)_ = 6.0, *p* = 1.4E-04; IgG4: *F*_(4, 124)_ = 9.7, *p* = 7.6E-07] and IgM [*F*_(4, 126)_ = 11.5, *p* = 5.4E-08; Figure 1], whereas the total levels of anti-α-syn IgG NAbs were similar in all groups, with neither age nor sex being significantly confounding factors. The relative levels of anti-α-syn IgG1 were higher in MSA compared to PD (*p* < 0.001; Figure 1B). Similarly, the anti-α-syn IgG3 levels were higher in MSA compared to PD (*p* = 0.008; Figure 1D). Anti-α-syn IgG2 levels described by disease group (*p* = 3.0E-04) were higher in PD compared to controls (*p* < 0.001; Figure 1C), as were the described anti-α-syn IgG4 levels by group (*p* = 3.5E-07), whereas anti-α-syn IgG4 was lower in PD compared to MSA and controls (controls: *p* = 0.005 and MSA: *p* < 0.001; Figure 1E). Further, anti-α-syn IgM levels were lower in both MSA and PD as compared to controls (MSA: *p* = 0.002 and PD: *p* = 0.001) described by group (*p* = 6.0E-04; Figure 1F).

Further, our model identified increased anti-Tau IgG1 levels in PD compared to MSA [*F*_(4, 125)_ = 5.0, *p* = 9.0E-04], also only described by group (*p* = 1.0E-03) with decreased levels in MSA compared to PD (*p* = 0.022; Figure S1). For detailed statistics of each parameter and adjusted *post hoc* Tukey analyses see Table S1.

### Global Plasma Antibody Concentrations

**Figure 2:**
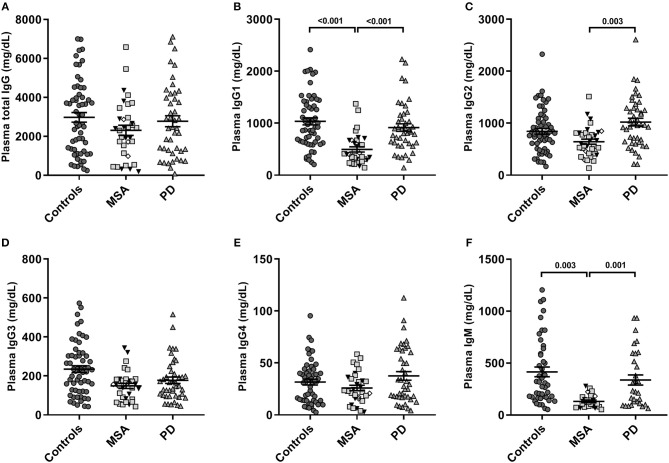
Global plasma antibody levels. Distribution of global plasma amounts (mg/dL) of antibodies in patients with Multiple System Atrophy (MSA, *n* = 34) divided into subtypes (MSA-P: gray squares, *n* = 20; MSA-C: black triangles, *n* = 11; MSA-C+P, *n* = 3: white rhombi), patients with Parkinson's disease (PD, *n* = 34), and controls (*n* = 59). Absolute plasma concentrations to ELISA of **(A)** total IgG, **(B)** IgG1, **(C)** IgG2, **(D)** IgG3, **(E)** IgG4, and **(F)** IgM antibodies. Dot plots show plasma Ig concentrations with mean values (horizontal bars) ± SEM. Differences were tested using one-way ANOVA and Tukey's *post hoc* test adjusted for age and sex.

The global antibody concentrations (total IgG, IgG1-4, and IgM) are shown in Table 2 and presented in Figure 2. Interestingly, we observed significantly lower global IgG1 levels [*F*_(4, 129)_ = 9.9, *p* = 5.2E-07] described by group (3.6E-04) in MSA compared to PD and controls (both *p* < 0.001; Figure 2B). The levels of IgG2 were also significantly lower [*F*_(4, 132)_ = 3.8, *p* = 6.0E-03] described by group (*p* = 3.0E-03) in MSA compared to PD patients (*p* = 0.003), but not compared to controls (*p* = 0.084; Figure 2C).

**Table 2:** Global plasma immunoglobulin levels.

	**Group**	**Amount (mg/dL)**	**Range**
Total IgG	Controls	2,978 ± 1,888	249.9–7,002
(mg/dL)	MSA	2,312 ± 1,511	191.6–6,585
	PD	2,776 ± 1,872	86.03–7,109
IgG1	Controls	1,037 ± 518	207.1–2,417
(mg/dL)	MSA	496 ± 291^§^^,^ ^≠^	145–1,375
	PD	916 ± 478^#^	143.7–2,232
IgG2	Controls	843 ± 422	169–2,328
(mg/dL)	MSA	645 ± 288^≠^	136.5–1,515
	PD	1,022 ± 483^#^	212–2,609
IgG3	Controls	235 ± 134	43–574
(mg/dL)	MSA	149 ± 75	43–344
	PD	177 ± 108	46–516
IgG4	Controls	31.7 ± 20.4	2.5–95.4
(mg/dL)	MSA	26.0 ± 15.1	2.8–58.5
	PD	37.6 ± 26.6	4.2–112.7
IgM	Controls	415 ± 316	53–1,204
(mg/dL)	MSA	131 ± 66^§^	54–278
	PD	337 ± 272^§^	63–933

Results are presented as mean ± standard deviation. MSA, Multiple System Atrophy; PD, Parkinson's disease.

§ Significantly different from controls.

# Significantly different from MSA.

≠ Significantly different from PD.

*For exact adjusted Tukey's post-hoc p-values, see Figure 1. For complete model statistics, see Table S2*.

Similar to IgG1 findings, there were significantly lower global IgM levels [*F*_(4, 95)_ = 6.6, *p* = 1.0E-04] described only by group (*p* = 3.0E-04) in MSA compared to PD (*p* = 0.001) and controls (*p* = 0.003; Figure 2F). Besides being described by group, the IgM levels were to a minor extent further described by sex [*F*_(1)_ = 6.80, *p* = 0.011], indicating sex as a confounding factor in IgM plasma levels. For detailed statistics see Table S2. Further, our model showed IgG3 levels to be significantly different [*F*_(4, 130)_ = 2.66, *p* = 0.035], however, this difference was not described by group, age, or sex. No significant differences were observed between the groups for IgG4 or total IgG levels.

### Total Plasma α-Synuclein

Total levels of plasma α-syn were significantly described by our model [*F*_(2, 125)_ = 7.46, *p* = 2.0E-05] described by group [*F*_(2)_ = 5.58, *p* = 5.0E-03] and were significantly lower in MSA [479 ± 175 ng/mL; (223–885 ng/mL)] compared only to controls [638 ± 251 ng/mL; (155–1285 ng/mL)] (*p* = 0.014; Figure S2). Total plasma α-syn levels were not significantly different in PD [532 ± 170 ng/mL; (236–984 ng/mL); controls: *p* = 0.243; MSA: *p* = 0.280].

### Correlation to Clinical Outcomes

The aberrant levels of those individual NAbs or global antibodies with significant described outcomes were correlated with disease duration and H & Y staging. The anti-α-syn IgM NAbs correlated significantly with disease duration in the PD group [Spearman's correlation (*rho*) = −0.389, *p* = 0.014; see Table S3]. Further, α-syn levels in plasma correlated negatively with disease duration in MSA (*rho* = −0.389, *p* = 0.028) and were positively associated with the H & Y staging in PD (*rho* = 0.338, *p* = 0.04; see Table S3).

## Discussion

To our knowledge, this is the first study exploring the global concentrations of plasma IgM, total IgG, and IgG1-4 subclasses along with subclass autoantibodies toward conventional neuropathological amyloidogenic proteins i.e., α-syn, pSer129-α-syn, β-syn, γ-syn, Aβ and tau protein, in MSA and PD patients compared to a control group. Our data suggest that the peripheral immune system is deregulated in MSA and PD, constrained mainly toward α-syn, that in aggregated form is the major pathological hallmark protein in MSA and PD.

We previously hypothesized that the relatively mild, but significant, decrease in high-affinity/avidity anti-α-syn NAbs in plasma of PD patients may parallel the relative slow progression of PD pathology compared to MSA, whereas the rapidly progressing clinical course of MSA could be related to the near absence of high-affinity/avidity anti-α-syn NAbs in that condition (27). Consequently, in this study, we have made a detailed investigation of the nature of the autoimmune decline in relation to IgM and IgG1-4 levels.

The main finding of this study is the significantly reduced anti-α-syn IgM levels in both MSA and PD patients compared to healthy controls. Remarkably, the anti-α-syn IgM levels were negatively correlated with disease duration in PD. Natural IgMs are evolutionarily-conserved proteins of the innate and adaptive immune system that react with a variety of epitopes expressed on both self- and non-self-antigens, which have previously been associated with autoimmune and inflammatory diseases (35). Natural IgMs are often the products of long-lived, self-renewing B-1 cell clones that arise during immune development without an absolute requirement for exogenous antigenic stimulation (36). IgMs lead to opsonization of antigens, are a potent activator of the classical complement pathway, and mediates endocytosis through Fcμ receptors on phagocytotic cells (37). Reduction of these peripheral IgM NAbs would be matched by lower levels of antigen-antibody complexes, thus reducing uptake of complexes by macrophages and other phagocytotic cells.

Different antibody isotypes carry out different functions, i.e., IgG but not IgM antibodies can penetrate tissue, where they activate complement and bind Fc receptors on resident macrophages and NK cells to induce antibody-dependent cellular response (38). Total levels of anti-α-syn IgG were the subject of several previous investigations, which yielded discordant results, as reviewed by Scott et al. (39). While there was no difference in total anti-α-syn IgG NAb levels between MSA, PD and control groups, we found several significant group differences in IgG subclass levels. Notably, we found significantly higher levels of anti-α-syn IgG1 and IgG3 in MSA compared to PD groups, increased levels of anti-α-syn IgG2 in PD compared to controls and decreased anti-α-syn IgG4 levels in PD compared to MSA and control groups. IgG1 and IgG3 are predominately activating antibodies of the classical complement pathway mediated by C1q (40). In neurodegenerative diseases such as AD and PD, it is known that accumulating pathological proteins elicit local pro-inflammatory activation and chronic responses of microglial activation, mediating clearance of the antigens through the complement system (40). One post mortem study showed complement activation to be associated with Lewy bodies in PD (41). An increase in pro-inflammatory activating autoantibodies could partly explain the microgliosis in MSA and PD patients (42–45).

Immunocomplexes are the direct, natural, and real-time products of an immune response and we can speculate at this point that a substantial number of anti-α-syn antibodies will be engaged by antigens and therefore not measurable in ELISA with α-syn coated plates, unless the samples were first mildly denatured to free up the antibodies. One possible explanation for the lower antibody levels is that the polyclonal NAbs are relatively selective for oligomers and aggregates but have lower affinity/avidity for monomeric antigen. Indeed, the α-syn immobilized on ELISA plates display heterogenous epitopes, i.e., both monomeric epitopes and the oligomeric/aggregated epitopes that are probably more attractive for polyclonal NAbs.

To evaluate the distinctiveness of the α-syn autoantibody patterns in MSA and PD, we have in this study also investigated levels of antibodies to certain proteins involved in other neurodegenerative diseases. We found that only the anti-tau IgG1 levels were increased in PD compared to MSA. A previous study has likewise shown alterations in anti-tau NAbs against for PD patients with dementia (46). Present results suggest that anti-tau and anti-Aβ NAbs do not play a major role in MSA, but could contribute to PD pathogenesis, especially in advanced or aggressive forms of the disease. These results suggest that the immune system is responding mainly toward the pathological hallmarks and not overall to amyloidogenic proteins.

β-syn shows weak aggregation properties, whereas γ-syn shows stronger aggregation properties, resembling those of α-syn (47). However, neither β-syn nor γ-syn have any established pathological relation in PD and MSA. Even though β-syn and γ-syn present potentially overlapping epitopes with α-syn due to their high amino acid sequence homology, 78% and 60%, respectively (48), we did not identify any aberrant findings for anti-β-syn or anti-γ-syn NAbs that survived correction for multiple comparison.

Global IgM and IgG levels have not hitherto been evaluated in MSA or PD. Notably, we found that the levels of global plasma IgM and IgG1 were significantly lower in MSA compared to PD and healthy control groups, and that global IgG2 levels were still lower in MSA compared to PD patients. These global antibody results may reflect systemic immunological differences in MSA and PD compared with healthy individuals and could furthermore indicate an especially decreased homeostatic capability or selective immunodeficiency in MSA, perhaps reflecting its more aggressive course.

At this point we can only speculate that the IgM producing B-1 cell population may already be reduced or impaired in pre–morbid MSA/PD individuals, resulting in an overload with toxic α-syn species in the nervous system putting a greater burden on the clearance mechanisms. In this scenario, after the initial recognition of toxic α-syn species, the autoantibody response changes to an IgG-dominated response characterized by lower-affinity/avidity antibodies, as supported by present results. This antigen-selective immunodeficiency in anti-α-syn NAbs can be linked to the distinct clinical phenotypes of PD and MSA, with MSA being much more rapidly progressing and having the more severe α-syn accumulation (49, 50).

Plasma levels of α-syn have been extensively evaluated in PD [reviewed in (39)], whereas only three studies have hitherto measured α-syn levels in plasma from MSA patients (51–53). We found that MSA patients had significantly lower plasma α-syn compared to controls, thus in agreement with our previous results (27) using similar methodology. However, this finding stands in contrast to previous studies based on traditional sandwich ELISAs, which suggested higher (51, 52) or unaltered (53) plasma α-syn levels in MSA patients compared to controls. We suppose that these discrepancies may be related either to methodological difference or cohort composition. Nonetheless, we observed that α-syn levels were negatively associated with disease duration in MSA, an association that was absent in PD. We suggest that the reason for the association in MSA could be due to a wider or faster neurodegeneration, probable due to α-syn aggregation. However, we identified a positive association between α-syn levels and disease severity defined by H & Y staging in PD patients in accordance with Lin et al. (54). This suggests that peripheral levels of α-syn may play a role in the pathogenesis of PD.

The study has some limitations. MSA is considered a rare disorder, therefore, we had only access to a limited number of MSA patients. Further, the patients were recruited out of single neurological unit, hence our results should be validated in a larger cohort, preferably in a longitudinal study. The two disease groups were similar regarding demographics, however, the control group included limitations regarding age and sex. These two factors may have an impact on the humoral Ig immune system, and even though we incorporated age and sex as confounding variables in our statistical models, this need to be considered when interpreting the results. Nonetheless, our study points to overlapping pathogenic mechanisms, as we identified important trends in the humoral immune system of MSA and PD patients.

In summary, we have found evidence for aberrant peripheral IgM and IgG subclass responses in MSA and PD patients, supporting involvement of an autoimmune component in these disorders. The disease-specific α-syn antibody reactive patterns suggest distinct processes of peripheral immune pathogenesis in MSA and PD, which are potentially useful for discriminative diagnosis. Our results indicate a reduced capacity for immune clearance and/or blocking of toxic α-syn transmission and propagation in these conditions, and further support the rationale for immunotherapy with high-affinity/avidity α-syn antibodies for treatment of MSA and PD.

## Data Availability

The datasets used and analyzed during the current study are available from the corresponding author on reasonable requests from a qualified researcher.

## Ethics Statement

This study was approved by The Danish National Committee on Health Research ethics, The Capital Region of Denmark, Kongens Vænge 2, DK-3400 Hillerød, Denmark (Protocol No.: H-1-5016-232). All participants gave written informed consent for inclusion in the biobank according to the World Medical Association Declaration of Helsinki.

## Author Contributions

JF and TB conceived and designed the study with critical input from RR, LP, SA, and BP. JF and TB wrote and BP, SA, and RR assisted in editing with input from all co-authors. SC and LP assisted in electrochemiluminescence assays and production of phosphorylated recombinant protein. AL, A-MH, LS, SB, and KW assisted in all clinical work e.g., recruitment of patients, follow-up diagnoses, and clinical data registry. All authors read and approved the final manuscript.

### Conflict of Interest

SC was employed by company H. Lundbeck A/S. The remaining authors declare that the research was conducted in the absence of any commercial or financial relationships that could be construed as a potential conflict of interest.
