# Supplementary material for: Distinct Autoimmune Anti-α-Synuclein Antibody Patterns in Multiple System Atrophy and Parkinson’s Disease
Source: Front Immunol. 2019 Sep 24;10:2253. doi: 10.3389/fimmu.2019.02253 (PMC6769034; doi:10.3389/fimmu.2019.02253)
Supplement: Supplementary file 1 [file Data_Sheet_1.PDF]

frontiers

Distinct autoimmune anti-α-synuclein antibody patterns in Multiple System Atrophy and Parkinson's Disease

Jonas Folke[1], Rasmus Rydbirk[1], Annemette Løkkegaard[2], Lisette Salvesen[2], Anne-Mette Hejl[2], Charlotte Starhof[2], Sára Bech[2], Kristian Winge[3,4], Søren Christensen[5], Lars Østergaard Pedersen[6], Susana Aznar[1], Bente Pakkenberg[1,7] and Tomasz Brudek[1,*].

[1]: Research Laboratory for Stereology and Neuroscience, Bispebjerg-Frederiksberg Hospital, University Hospital of Copenhagen, Nielsine Nielsens Vej 6B, DK-2400, Copenhagen, Denmark.
[2]: Department of Neurology, Bispebjerg-Frederiksberg Hospital, University Hospital of Copenhagen, Ebba Lunds Vej 44, DK-2400, Copenhagen, Denmark.
[3]: Department of Neurology, Zealand University Hospital, Vestermarksvej 11, DK-4000, Roskilde, Denmark
[4]: Bispebjerg Movement Disorders Biobank, Bispebjerg-Frederiksberg Hospital, University Hospital of Copenhagen, Nielsine Nielsens Vej 6B, DK-2400, Copenhagen, Denmark.
[5]: H. Lundbeck A/S, Ottiliavej 9, DK-2500, Valby, Denmark.
[6]: Department of Immunology and Microbiology, Faculty of Health, University of Copenhagen, Blegdamsvej 3B, DK-2200, Copenhagen, Denmark.
[7]: Institute of Clinical Medicine, Faculty of Health, University of Copenhagen, Blegdamsvej 3B, DK-2200, Copenhagen, Denmark.
[#]: Correspondence to:

Research Laboratory for Stereology and Neuroscience
Bispebjerg-Frederiksberg Hospital, University Hospital of Copenhagen
Nielsine Nielsens Vej 6B, stair 11B, 2nd Floor
DK-2400, Copenhagen, Denmark
Tel. +45 38 63 61 13
Fax. +45 38 63 98 22
E-mail: Tomasz.brudek@regionh.dk

1 Supplementary Figures and Tables

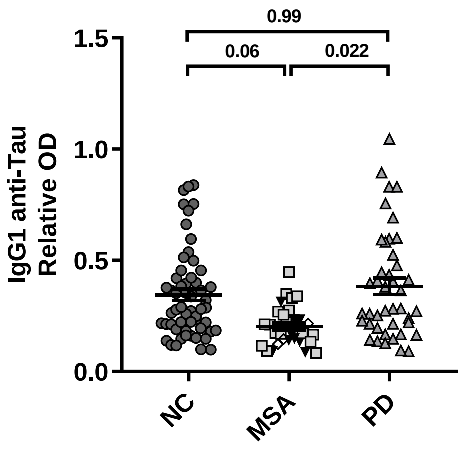

Figure S1 Plasma Tau naturally occurring autoantibody IgG1 levels. Distribution of relative anti-α-synuclein naturally occurring autoantibody plasma levels in patients with Multiple System Atrophy (MSA, n=34) divided into subtypes (MSA-P: grey squares, n=20; MSA-C: black triangles, n=11; MSA-C+P, n=3: white squares), patients with Parkinson's disease (PD, n=34), and controls (NC, n=59). ELISA relative ODs of anti-Tau IgG1 autoantibodies. Dot plots show relative ODs with mean values (horizontal bars) ± SEM. Differences were one-way ANOVA and Tukey's post hoc test adjusted for age and sex.

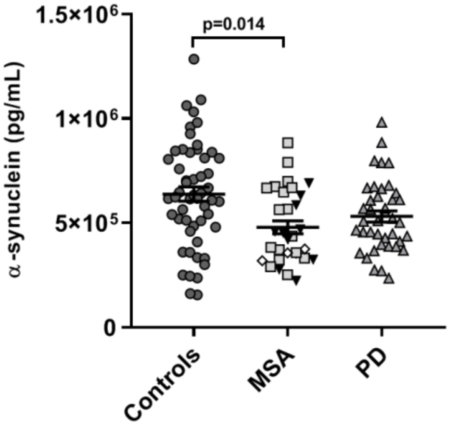

Figure S2 Total plasma α-synuclein amounts. Distribution of plasma content (pg/dL) of total α-synuclein in patients with Multiple System Atrophy (MSA, n=34), divided into subtypes (MSA-P: grey squares, n=20; MSA-C: black triangles, n=11; MSA-C+P, n=3: white rhombi), patients with Parkinson's disease (PD, n=34), and controls (n=59). Indicated as absolute plasma concentrations to U-PLEX® Human α-syn Kit of α-synuclein. Dot plots show plasma α-synuclein concentrations with mean values (horizontal bars) ± SEM. Differences were tested using one-way ANOVA and Tukey's *post hoc* test adjusted for age and sex.

Table S1: Statistical comparison of plasma concentrations of autoantibody subclasses for neurodegenerative pathological proteins

**Antigen**	**Model statistics**			**Groups**	**Age**	**Sex**	**Tukey Post hoc test (p-values)**		
**α-synuclein**	**p-values**	R^2	**F-statistics**	**p-values**	**p-values**	**p-values**	**Controls vs MSA**	**Controls vs PD**	**MSA vs PD**
IgG1	**3.9E-07**	0.25	F(4,109)=10.3	**6.9E-06***	0.055	0.936	0.070	0.140	**<0.001***
IgG2	**5.6E-06**	0.20	F(4,114)=8.4	**3.0E-04***	0.889	0.740	0.059	**<0.001***	0.111
IgG3	**1.4E-04**	0.13	F(4,120)=6.0	**9.0E-03***	0.070	0.826	0.110	0.835	**0.008***
IgG4	**7.6E-07**	0.21	F(4,124)=9.7	**3.5E-07***	0.445	0.815	0.165	**0.005***	**<0.001***
IgG-Total	0.067	0.03	F(4,130)=2.3	0.130	0.144	0.275	-	-	-
IgM	**5.4E-08**	0.24	F(4,126)=11.5	**6.0E-04***	0.900	0.070	**0.002***	**0.001***	0.999
**P-α-synuclein**									
IgG1	0.210	0.02	F(4,122)=1.5	0.070	0.269	0.768	-	-	-
IgG2	0.123	0.03	F(4,122)=1.9	0.763	0.219	0.077	-	-	-
IgG3	0.100	0.03	F(4,122)=2.0	0.897	0.255	0.377	-	-	-
IgG4	0.369	0.003	F(4,122)=1.1	0.813	0.440	0.138	-	-	-
IgG-Total	0.886	-0.02	F(4,122)=0.3	0.812	0.821	0.327	-	-	-
IgM	0.073	0.04	F(4,122)=2.2	0.192	0.484	0.328	-	-	-
**β-synuclein**									
IgG1	**0.010[#]**	0.08	F(4,111)=3.5	**0.020**	0.214	0.614	-	-	-
IgG2	0.343	0.004	F(4,123)=1.1	0.594	**0.044**	0.670	-	-	-
IgG3	**1.4E-03[#]**	0.11	F(4,119)=4.8	**7.0E-03**	**0.008**	**0.012**	-	-	-
IgG4	0.155	0.02	F(4,124)=1.7	0.617	**0.027**	0.231	-	-	-
IgG-Total	0.758	-0.02	F(4,127)=0.5	0.743	0.297	0.385	-	-	-
IgM	0.039[#]	0.05	F(4,128)=2.6	0.066	0.750	0.142	-	-	-
**γ-synuclein**									
IgG1	0.220	0.02	F(4,116)=1.5	0.094	0.437	0.420	-	-	-
IgG2	0.129	0.03	F(4,118)=1.8	0.221	0.056	0.073	-	-	-
IgG3	**0.037[#]**	0.05	F(4,121)=2.6	**0.018**	0.142	0.382	-	-	-
IgG4	0.133	0.03	F(4,121)=1.8	0.632	0.148	0.112	-	-	-
IgG-Total	0.197	0.02	F(4,126)=1.5	0.138	0.552	0.539	-	-	-
IgM	0.016	0.06	F(4,130)=3.2	0.158	0.552	0.070	-	-	-
**Tau**									
IgG1	**9.0E-04**	0.11	F(4,125)=5.0	**1.0E-03***	0.920	0.363	0.060	0.997	**0.022***
IgG2	0.136	0.02	F(4,130)=1.8	0.756	0.249	0.119	-	-	-
IgG3	**9.0E-03[#]**	0.07	F(4,129)=3.6	0.228	**0.027**	**0.011**	-	-	-
IgG4	**7.0E-03[#]**	0.04	F(4,126)=3.7	0.135	0.100	0.130	-	-	-
IgG-Total	0.606	0.009	F(4,125)=0.7	0.321	0.447	0.479	-	-	-
IgM	0.111	0.03	F(4,127)=1.9	0.997	0.645	**0.034**	-	-	-
**Amyloid β-peptide**									
IgG1	0.633	-0.01	F(4,125)=0.6	0.456	0.456	0.248	-	-	-
IgG2	0.288	0.008	F(4,133)=1.3	0.888	0.084	0.815	-	-	-
IgG3	0.586	-0.01	F(4,122)=0.7	0.420	0.115	0.837	-	-	-
IgG4	0.267	0.01	F(4,126)=1.3	0.141	0.147	0.567	-	-	-
IgG-Total	**0.045[#]**	0.001	F(4,120)=2.5	**0.026**	**0.010[#]**	0.199	-	-	-
IgM	0.094	0.03	F(4,121)=2.0	0.159	0.934	0.767	-	-	-

MSA: Multiple system atrophy. PD: Parkinson's disease. The F(x,y) represents the model F ratio statistics where x=degrees of freedom to the group and y=degrees of freedom for error variance. P-values for multiple regression analyses identifying significant predicted outcomes in a model including disease group, age and sex. Significant model statistics cut-off where set at p=0.001 after Bonferroni correction. [#]: did not pass multiple correction. *: Significant predicted variable outcomes.

Table S2 Statistical comparison of global plasma antibody subclasses

Antigen	Model statistics			Groups	Age	Sex	Tukey Post hoc test (p-values)		
**Global antibodies**	**p-values**	R^2	**F-statistics**	**p-values**	**p-values**	**p-values**	**Controls vs MSA**	**Controls vs PD**	**MSA vs PD**
IgG1	**5.2E-07***	0.21	F(4,129)=9.9	**3.6E-04***	0.713	0.878	**<0.001**	0.874	**<0.001**
IgG2	**6.0E-03***	0.08	F(4,132)=3.8	**3.0E-03***	0.183	0.870	0.751	0.084	**0.003**
IgG3	**0.035***	0.05	F(4,130)=2.7	0.117	0.880	0.331	-	-	-
IgG4	0.152	0.02	F(4,123)=1.7	0.175	0.713	0.253	-	-	-
IgG-Total	0.177	0.02	F(4,132)=1.6	0.530	0.076	0.763	-	-	-
IgM	**1.0E-04***	0.18	F(4,95)=6.6	**3.0E-04***	0.588	**0.011***	**0.003**	0.934	**0.001**

MSA: Multiple system atrophy. PD: Parkinson's disease. The F(x,y) represents the model F ratio statistics where x=degrees of freedom to the group and y=degrees of freedom for error variance. P-values for multiple regression analyses identifying significant predicted outcomes in a model including disease group, age and sex. *: significant predicted outcome p<0.05.

Table S3 Correlation analyses of significant outcomes

	MSA				PD			
	**Disease duration**		**Hoehn & Yahr**		**Disease duration**		**Hoehn & Yahr**	
	r	p-value	*r*	p-value	*r*	p-value	*r*	p-value
Anti-α-syn-IgG1	0.12	0.512	-0.23	0.248	-	-	-	-
Anti-α-syn-IgG2	-	-	-	-	0.03	0.851	-0.18	0.270
Anti-α-syn-IgG4	-	-	-	-	-0.19	0.276	-0.09	0.614
Anti-α-syn-IgM	0.11	0.560	0.002	0.993	-0.39	**0.014***	-0.19	0.234
Global Anti- IgG1	0.28	0.119	0.25	0.226	-	-	-	-
Global Anti- IgM	-0.34	0.103	-0.009	0.97	-	-	-	-
Total α-syn	-0.39	**0.028***	0.32	0.132	0.10	0.55	0.338	**0.041***

*All correlations were performed using Spearman's correlation. *: significant p-values <0.05.*
